# Comment on Alfageme-Garcia et al. Respectful Children’s Shoes: A Systematic Review. *Children* 2024, *11*, 761

**DOI:** 10.3390/children11101210

**Published:** 2024-09-30

**Authors:** Gabriel Gijon-Nogueron

**Affiliations:** 1 Department of Nursing and Podiatry, University of Malaga, 29071 Malaga, Spain; gagijon@uma.es; 2 IBIMA Plataforma BIONAD, 29010 Malaga, Spain

I recently received an alert regarding the publication of the article [1] in the journal *Children*.

As this topic falls within my field of research and considering the proliferation of various theories about the use of minimalist or so-called respectful footwear, I have thoroughly reviewed this piece of work. First, I would like to congratulate the authors on addressing such an interesting topic. However, as a fellow scientist, I feel compelled to bring to your attention some serious methodological errors in the article, which I have detailed as follows:

1. The Results table includes two bachelor theses that neither present clear results nor can be considered research work.
2. Another article [2] discusses about the influences on children’s choice of footwear, their perception of comfort, and the language they use to describe footwear experiences, which should have been excluded from this study about minimalist footwear.
3. The inclusion of a Delphi agreement about therapeutic footwear is questionable; while it can be analyzed, it should not have been included as it does not provide results according to the objectives of this systematic review [3].

I consider the publication of the aforementioned information potentially harmful and threatens the reliability of the attained results and conclusions. It may introduce bias in the opinions of both researchers and clinicians who are unable to critically assess the content of this review.

Furthermore, the methodology is highly deficient. The inclusion criteria are poorly designed, the PICO question is incorrectly framed in the exclusion criteria, and I am sure that the first two articles do not appear in the databases searched for this review. A majority of the references are books that do not have supporting research work, in addition to being very old, thus the evolution of the information may be outdated. There are numerous issues which make me question the reviewers’ qualifications, as these are fundamental errors and cannot be permitted by your journal.

Lastly, the conclusions are not based on the results of the analyzed articles; they are mere speculations that have been presented in a random and biased manner.
